# Factors that affect the development of acute hemorrhagic rectal ulcer syndrome and rebleeding

**DOI:** 10.1002/deo2.184

**Published:** 2022-11-22

**Authors:** Natsumi Uehara, Kazuaki Inoue, Yuichiro Kuroki, Naoki Miyao, Kenta Iwahashi, Reika Suzuki, Toshiyuki Endo, Kunio Asonuma, Erika Yoshida, Naoko Koshibu, Akihiro Tabuchi, Misako Tohata, Shotaro Hanamura, Kuniyo Gomi, Yorimasa Yamamoto, Masatsugu Nagahama

**Affiliations:** ^1^ Department of Gastroenterology Medical Topia Soka Hospital Saitama Japan; ^2^ Department of Gastroenterology Showa University Fujigaoka Hospital Kanagawa Japan; ^3^ Department of Gastroenterology International University of Health and Welfare Narita Hospital Chiba Japan; ^4^ Department of Gastroenterology St. Marianna University School of Medicine Yokohama City Seibu Hospital Kanagawa Japan; ^5^ Department of Internal medicine Honda Hospital Tokyo Japan; ^6^ Department of Gastroenterology Kikuna Memorial Hospital Kanagawa Japan

**Keywords:** endoscopy, hemorrhage, hemostasis, rectum, ulcer

## Abstract

**Objectives:**

Acute hemorrhagic rectal ulcer syndrome (AHRUS) causes massive bleeding and often recurrent rebleeding from rectal ulcers that form immediately above the dentate line. This study aimed to determine the clinical background and risk factors contributing to rebleeding in patients with AHRUS and the most appropriate method of hemostasis treatment.

**Methods:**

This retrospective study included 93 patients diagnosed with AHRUS at Showa University Fujigaoka Hospital, Japan, between April 2009 and November 2018. Information on clinical background factors, endoscopic findings, and hemostasis was obtained from medical records. The relationship with episodes of rebleeding was analyzed by multivariate logistic regression analysis.

**Results:**

The median age was 79 years, and 84 patients (90%) had a performance status of grade 2 or higher. The patients had multiple background factors, with a median number of 5 per patient. The background factors could be classified into two major factors: those related to arteriosclerosis and those related to delayed wound healing.

In the multivariate analysis, significantly more rebleeding occurred in patients with active bleeding during the initial endoscopy (odds ratio 4.88, 95% confidence interval 1.80–14.46, *p* = 0.003); significantly less rebleeding occurred in patients for whom hemostasis was first performed by clipping (odds ratio 0.30, 95% confidence interval 0.09–0.88, *p* = 0.035).

**Conclusions:**

In bedridden older individuals with poor general health, multiple combinations of arteriosclerosis‐related factors and protracted wound healing factors can induce AHRUS. We strongly recommend performing hemostasis via the clipping method on suspected bleeding points, including active bleeding sites, in AHRUS.

## INTRODUCTION

Acute hemorrhagic rectal ulcer syndrome (AHRUS) is characterized by massive bleeding from the rectum in older patients with comorbidities. It was first reported by Kono et al. in 1980.^1^ Hirooka established the clinical entity of AHRUS based on his experience gained from treating 10 patients in 1984.^2^ This disease is characterized by the sudden onset of painless but extensive bleeding from rectal ulcers located at the distal rectum, directly above the dentate line, in patients suffering from severe comorbidities.^3^

There have been many reports of AHRUS in Japan^3^, ^4^, ^5^, ^6^ and some recent reports from other Asian countries.^7^, ^8^, ^9^ Rectal bleeding from a Dieulafoy lesion has been reported in studies from western countries.^10^, ^11^ A significant degree of variability exists in the number of reported AHRUS cases worldwide; these discrepancies may depend on how widely this clinical entity is recognized.

In 1997, Nakamura et al. analyzed 50 cases and observed that arteriosclerosis and a bedridden state were closely related to AHRUS^5^; no other associated factors were reported. AHRUS is also associated with high rebleeding rates (24%–50%)^12^, ^13^, ^14^, ^15^, ^16^ and mortality (20%–47.2%) following its onset.^12^, ^16^ Although it is concerning that the general condition of patients with AHRUS worsens due to repeated bleeding, no studies have been conducted to determine the most appropriate endoscopic hemostatic method. Therefore, in this study, we aimed to identify the clinical background and risk factors associated with rebleeding and determine the most appropriate method of hemostasis for AHRUS treatment.

## METHODS

### Position of AHRUS at Showa University Fujigaoka Hospital, Japan

**FIGURE 1:**
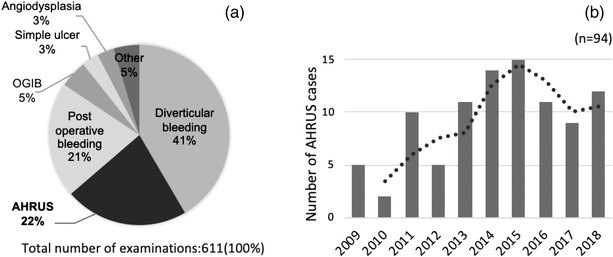
Acute hemorrhagic rectal ulcer syndrome (AHRUS) at the Showa University Fujigaoka Hospital from April 2009 to October 2018. (a) Among the diagnoses based on the 611 emergency colonoscopies for lower gastrointestinal bleeding, 136 (22%) were AHRUS, the second most common cause of bleeding. (b) Annual trends in the number of AHRUS patients; although there are variations, the trend was broadly increasing. Abbreviation: OGIB, obscure gastrointestinal bleeding

Approximately 10,000 endoscopic examinations are conducted annually at Showa University Fujigaoka Hospital, an acute care general hospital in Japan. From April 2009 to October 2018, there were 611 emergency endoscopic examinations for lower gastrointestinal bleeding conducted here, of which 136 (22%) were caused by AHRUS (Figure 1a). During the same period, 94 patients were diagnosed with AHRUS at the hospital; this number generally increased over time (Figure 1b).

### Participants

Ninety‐four patients were diagnosed with AHRUS at the hospital between April 2009 and October 2018; 93 were included in this study, and one was excluded due to missing data. We retrospectively analyzed the patients’ medical records and endoscopic files.

### Survey items

We examined clinical background factors, lesion factors, and therapeutic factors. We extracted the background factors: gender, age, bedridden state, antithrombotic therapy, cardiac disease, infection, diabetes mellitus, cerebrovascular disease, on dialysis, cancer, fracture, hypoalbuminemia, and corticosteroid usage. All parameters were evaluated based on the data obtained immediately before onset.

**FIGURE 2:**
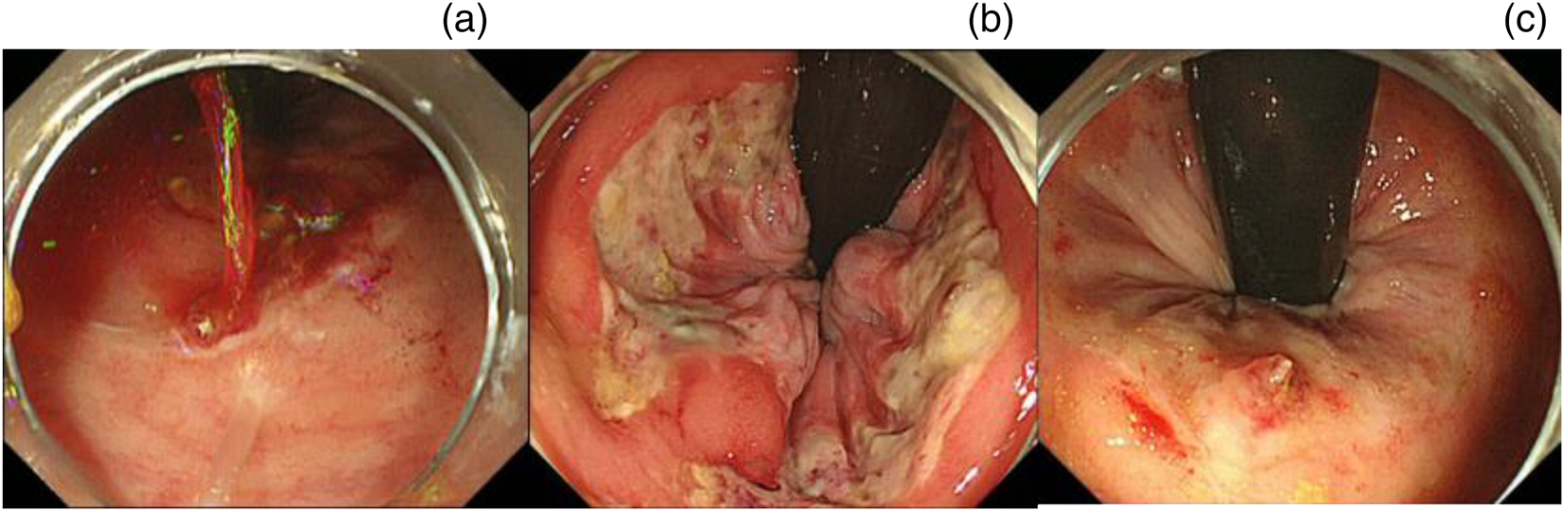
Case endoscopic photographs of lesion morphology of acute hemorrhagic rectal ulcer syndrome. (a) A case of active bleeding. (b) A case of an ulcer floor‐forming lesion. (c) A case of a Dieulafoy lesion

We targeted lesion findings and endoscopic treatment at the time of initial endoscopy performed within 24 hours of the onset of bloody stool. Currently, there has been no established classification by lesion type of AHRUS. However, endoscopic findings have classified the lesions as circumferential ulcers, geographic‐shaped ulcers, round ulcers, and Dieulafoy lesions.^2^, ^17^, ^18^ In this study, the endoscopic findings were simplified based on the lesion activity and simplified morphology, as emergency endoscopy did not always provide sufficient observations. Therefore, the activity was classified based on the presence or absence of active bleeding (Figure 2a), and the morphology was classified as an ulcer floor‐forming lesion (Figure 2b) or a Dieulafoy lesion (Figure 2c).

Therapeutic factors were classified as follows: clipping method (CLIP), electrocoagulation with single‐use electrosurgical hemostatic forceps (EC), argon plasma coagulation, endoscopic band ligation, or combinations of CLIP and EC methods. The definition of each factor is presented in Table 1. Rebleeding was defined as new bleeding requiring endoscopy within 30 days of the last bleeding, after which it was counted as a new case.

**TABLE 1:** Definitions of patients’ backgrounds, lesions, and therapeutic factors

**Clinical background factors**	
Bedridden state	Bedridden for more than half a day, equivalent to the performance status (PS; Eastern Cooperative Oncology Group, USA) of grade 2 or higher
Antithrombotic therapy	Administration of anticoagulants or/and antiplatelet drugs
Cardiac disease	Heart failure, valvular disease, arrhythmia, and ischemic heart disease
Infection	Infections requiring therapeutic intervention
Diabetes mellitus	Diabetes requiring therapeutic intervention
Cerebrovascular disease	Symptomatic cerebrovascular disease
On dialysis	Patients on dialysis (any dialysis method)
Cancer	Not cured cancer regardless of the treatment and stage
Fracture	Fracture causing instrumental activities of daily living disability
Hypoalbuminemia	Serum albumin concentration below 2 g/ml
Corticosteroid usage	Regular or intermittent use of corticosteroids
Lesion factors	
Active bleeding	Active bleeding at the initial endoscopic examination
Dieulafoy lesion (D)	Dieulafoy ulcer (abnormally large, exposed vessels with small shallow ulcers) or vascular lesion (no ulcers but only exposed blood vessels)
Ulcer floor‐forming lesion (UL)	Any shape of lesions with ulcer floor
Therapeutic factors	
Clipping method (CLIP)	Endoscopic hemostasis using a clip (EZ clip; Olympus, Tokyo, Japan)
Electrocoagulation (EC)	Electrocoagulation with single‐use electrosurgical hemostatic forceps (Coagrasper; Olympus) and an electrosurgical generator (ESG‐100; Olympus)
CLIP + EC	Hemostasis in combination with CLIP and EC
Argon plasma coagulation (APC)	Argon plasma coagulation is a non‐contact coagulation technique using ionized argon gas (APC300/ICC200; Erbe, Tuebingen, Germany)
Endoscopic band ligation (EBL)	The bleeding point is pulled into the device, the elastic O‐ring is released, and the bleeding point is tied with the ring (Pneumo‐Activate EVL device; Sumitomo Bakelite, Tokyo, Japan)
Observation	Endoscopic hemostasis could not be performed because no exposed blood vessels were suspected of bleeding points or active bleeding.

### Statistical analysis

All statistical analyses were performed using R Commander (John Fox; McMaster University, Hamilton, Ontario, Canada), a graphical user interface for the R software environment (The R Foundation for Statistical Computing, Vienna, Austria). This analysis was conducted to identify the independent risk factors for rebleeding with AHRUS. Fisher's exact test was used for the univariate analysis. In the multivariate analysis, we excluded factors with values below 10 and those that showed multicollinearity according to the Akaike information criterion, after which logistic regression analysis was performed.

### Research ethics

After the institutional review board of Showa University approved this study (approval number: F2018C79), information on the research, including the purpose of materials and information use, was disclosed to the research participants. In addition, an opt‐out document was published on the portal website for clinical research; it was announced that the data would not be used if requested by the research participants.

## RESULTS

**FIGURE 3:**
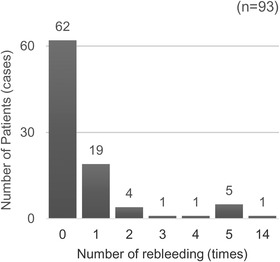
The number of rebleeding episodes per case. Of the 93 cases, 31 (33%) experienced rebleeding episodes more than once

All patients had comorbid AHRUS while hospitalized to treat their primary disease. Of the 93 patients included, 31 (33%) experienced rebleeding more than once. The highest frequency of rebleeding was 14 episodes in one patient (Figure 3). The survival prognosis for 30‐day mortality was 15.0% (14 cases), and 60‐day mortality was 17.2% (16 cases). In one patient, the direct cause of death was bleeding from AHRUS, whereas death was due to the primary disease in the remaining 15 patients.

### Clinical background factors

**FIGURE 4:**
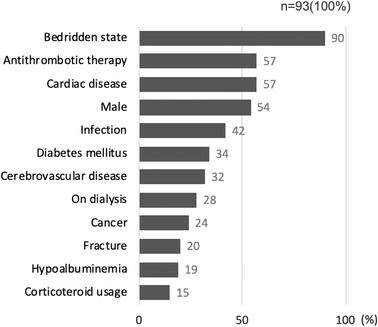
Patient prevalence for each background factor

The male‐to‐female ratio was 1.16:1, and the median age was 79 years (interquartile range [IQR] 51–83 years). Eighty‐four patients (90%) were bedridden, 53 were receiving antithrombotic therapy, 53 had cardiac diseases, 39 had infections, 34 had diabetes mellitus, 30 were diagnosed with cerebrovascular disease, 26 were on dialysis, 22 had cancer, 19 had a fracture, 18 had hypoalbuminemia, and 14 were corticosteroid users (Figure 4). Eighty‐seven patients (94%) exhibited multiple patient background factors. The median number of background factors per patient was 5 (IQR 3–6 factors).

### Lesion factors

Regarding the lesion type, ulcer floor‐forming lesions were observed in 77 patients, and Dieulafoy lesions were observed in 16 patients. In addition, active bleeding occurred at the initial endoscopy in 40 patients (43%).

### Therapeutic factors

**FIGURE 5:**
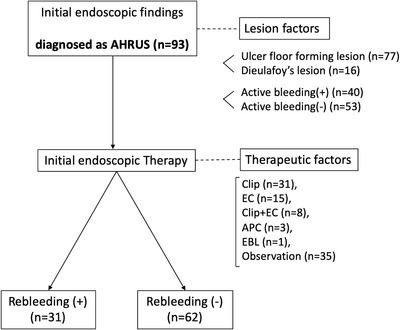
Flowchart of the result. Abbreviations: AHRUS, Acute hemorrhagic rectal ulcer syndrome; APC, argon plasma coagulation; CLIP, clipping; EBL, endoscopic band ligation; EC, electrocoagulation

Hemostatic procedures were performed in 58 patients (62%) at the initial endoscopy. Of these patients, CLIP^19^ was employed in 31, EC^20^ in 15, CLIP with EC in eight, argon plasma coagulation^21^ in three, and endoscopic band ligation^22^, ^23^ in one. No bleeding points were confirmed in the remaining 35 patients who had opted for observation, but rebleeding was observed in 11 patients (31.4%; Table 2). The results are summarized in the flowchart (Figure 5).

**TABLE 2:** Characteristics of the 11 patients with rebleeding of patients who had opted for observation at the first endoscopy

Patient (age, sex)	Lesion type	Re‐bleeding (times)	Treatment at the second endoscopy	Active bleeding after the second endoscopy	Prognosis(Cause of death)
82, M	UL	5	Observation	–	Death (bleeding with AHRUS)
81, F	UL	1	Ad. Gauze Tampon	–	Recovery
63, M	UL	5	CLIP	(+)	Recovery
61, M	UL	1	EC	–	Recovery
82, F	UL	1	EC	(+)	Recovery
79, F	UL	5	EC	(+)	Death (primary disease)
87, M	UL	5	Observation	–	Recovery
64, F	UL	1	Observation	–	Recovery
82, F	UL+D	2	Observation	(+)	Recovery
80, M	D	1	Observation	–	Death (primary disease)
70, M	UL	1	Observation	–	Recovery

Abbreviations: Ad. Gauze Tampon, treatment by stuffing gauze tampons soaked in diluted adrenaline solution into the anus to attempt pressure hemostasis; AHRUS, acute hemorrhagic rectal ulcer syndrome; CLIP, clipping; EC, electrocoagulation; D, Dieulafoy lesion; UL, Ulcer floor‐forming lesion.

### Univariate analysis

In the univariate analysis, none of the background factors showed significant differences between the rebleeding and non‐rebleeding groups. However, the number of patients with active bleeding at the initial endoscopy was significantly higher in the rebleeding group (rebleeding rate: 47.5% [19/40 patients], odds ratio [OR] 3.05, 95% confidence interval [CI] 1.16–8.36, *p* = 0.015). As for lesion types, the presence of Dieulafoy lesions and ulcer floor‐forming lesions was not significantly different between both groups. The rebleeding rate after initial endoscopic hemostasis was lower with CLIP (25.8%; 8/31 patients) than with EC (46.7%; 7/15 patients). However, both groups had no significant differences in therapeutic factors (Table 3).

**TABLE 3:** Univariate analysis of the presence or absence of rebleeding in cases of acute hemorrhagic rectal ulcer syndrome

Factors	Rebleeding (+) (*n* = 31) (%)	Rebleeding (–) (*n* = 62) (%)	Total (*n* = 93) (%)	Univariate OR (95% CI), *p‐*value
Age (median [IQR])	78 (58–82)	79 (51–85)	79 (51–83)	
Clinical background factors				
Bedridden state	29 (93.4)	55 (88.7)	84 (90.3)	1.83 (0.32–19.20), 0.71
Antithrombotic therapy	18 (58.1)	35 (56.5)	53 (57.0)	1.07 (0.41–2.82), 1.00
Cardiac disease	18 (58.1)	35 (56.5)	53 (57.0)	1.07 (0.41–2.82), 1.00
Male	18 (58.1)	32 (51.6)	50 (53.8)	1.29 (0.50–3.42), 0.66
Infection	16 (51.6)	23 (37.1)	39 (41.9)	1.80 (0.69–4.74), 0.19
Diabetes mellitus	11 (35.5)	23 (37.1)	34 (36.6)	0.93 (0.34–2.49), 1.00
Cerebrovascular disease	09 (29.0)	21 (33.9)	30 (32.3)	0.80 (0.27–2.22), 0.81
On dialysis	10 (32.3)	16 (25.8)	26 (28.0)	1.36 (0.47–3.85), 0.62
Cancer	07 (22.6)	15 (24.2)	22 (23.7)	0.91 (0.28–2.79), 1.00
Fracture	05 (16.1)	14 (22.6)	19 (20.4)	0.66 (0.17–2.23), 0.59
Hypoalbuminemia	06 (19.4)	12 (19.4)	18 (19.4)	1.00 (0.27–3.30), 1.00
Corticosteroid usage	07 (22.6)	7(11.3)	14 (15.1)	2.27 (0.61–8.53), 0.22
Lesion factors				
Ulcer floor‐forming lesion	25 (80.6)	52 (83.9)	77 (82.8)	0.80 (0.23–3.01), 0.77
Dieulafoy lesion	06 (19.4)	10 (16.1)	16 (17.2)	1.24 (0.33–4.30), 0.77
Active bleeding	19 (61.3)	21 (33.9)	40 (43.0)	3.05 (1.16–8.36), 0.015*
Therapeutic factors				
CLIP	08 (25.8)	23 (37.1)	31 (33.3)	0.59 (0.20–1.66), 0.35
EC	07(22.6)	08 (12.9)	15 (16.1)	1.95 (0.54–6.98), 0.25
CLIP + EC	03 (9.7)	05 (8.1)	08 (8.6)	NE
APC	02 (6.5)	01 (1.6)	03 (3.2)	NE
EBL	00 (0.0)	01 (1.6)	01 (1.1)	NE
Observation	11 (35.5)	24 (38.7)	35 (37.6)	0.87 (0.32–2.32), 0.82

*: *p* < 0.05.

Abbreviations: 95% CI, 95% confidence interval; APC, argon plasma coagulation; CLIP, clipping; EBL, endoscopic band ligation; EC, electrocoagulation; IQR, interquartile range; NE, not evaluated; OR, odds ratio.

### Multivariate analysis

In the multivariate analysis, active bleeding at the initial endoscopy had a significantly higher risk for rebleeding (OR 4.88, 95% CI 1.80–14.46, *p* = 0.003). Conversely, hemostasis performed using CLIP at the initial endoscopy had a significantly lower risk for rebleeding (OR 0.30, 95% CI 0.09–0.88, *p* = 0.035; Table 4). The breakdown of treatment methods according to the presence or absence of active bleeding is summarized in Table 5.

**TABLE 4:** Multivariate analysis of the presence or absence of rebleeding in acute hemorrhagic rectal ulcer syndrome cases

Factors	Rebleeding (+) (*n* = 31) (%)	Rebleeding (–) (*n* = 62) (%)	Total (*n* = 93) (%)	Multivariate OR (95% CI), *p‐*value
Active bleeding	19 (61.3)	21 (33.9)	40 (43.0)	**4.88 (1.80–14.46), 0.003** **
CLIP	08 (25.8)	23 (37.1)	31 (33.3)	**0.30 (0.09–0.88), 0.035** *

* : *p* < 0.05.

** : *p* < 0.01.

Abbreviations: 95% CI, 95% confidence interval; CLIP: clipping; OR, odds ratio.

**TABLE 5:** Breakdown of therapeutic factors by active bleeding and rebleeding in acute hemorrhagic rectal ulcer syndrome

[Active bleeding (+)]	Rebleeding (+) (n = 19)	Rebleeding (−) (n = 21)	Total (n = 40)
CLIP	8	13	21
EC	5	2	7
CLIP+EC	3	5	8
APC	2	0	2
EBL	0	1	1
Observation	1	0	1

[Active bleeding (−)]	Rebleeding (+) (n = 12)	Rebleeding (−) (n = 41)	Total (n = 53)
CLIP	0	10	10
EC	2	6	8
CLIP+EC	0	0	0
APC	0	1	1
EBL	0	0	0
Observation	10	24	34

Abbreviations: CLIP, clipping; EC, electrocoagulation; APC, argon plasma coagulation; EBL, endoscopic band ligation.

## DISCUSSION

### Underlying disease

Several studies have reported that AHRUS develops in bedridden patients with comorbidities.^3^, ^4^, ^5^, ^6^, ^8^, ^13^, ^15^, ^24^ Nakamura et al. demonstrated via the laser‐Doppler method that only patients with AHRUS had decreased lower rectal blood flow while in the supine position,^3^ which the clinical history of arteriosclerosis can explain.^5^, ^12^, ^13^

This study observed a median of five clinical background factors per patient. Frequently observed background factors were arteriosclerosis‐related,^5^, ^12^, ^13^ such as antithrombotic therapy, cardiac disease, diabetes mellitus, cerebrovascular disease, on dialysis, and protracted wound healing factors^25^ including infection, diabetes mellitus, cancer, hypoalbuminemia, and corticosteroid usage. In older individuals with multiple comorbidities and poor general health, hypoperfusion of the lower rectum (due to arteriosclerosis and a bedridden state), arteriosclerosis‐related factors, and protracted wound healing factors are expected to be closely related to AHRUS development.

In this study, the 30‐day mortality rate was 15.0%. The direct cause of death in most AHRUS patients was not bleeding from AHRUS but rather their primary disease. In such cases, AHRUS is only one of the signs of death; it might be challenging to save the patients even if we succeeded in hemostasis. On the other hand, nearly 80% of patients had successful endoscopic hemostasis and their general condition was restored.

Although further studies are needed to determine whether controlling rebleeding improves overall survival in AHRUS patients, preventing rebleeding with AHRUS, which is one of the factors that worsen patients’ general conditions, is crucial to recovery.

### Reasons for the high incidence of rebleeding

**FIGURE 6:**
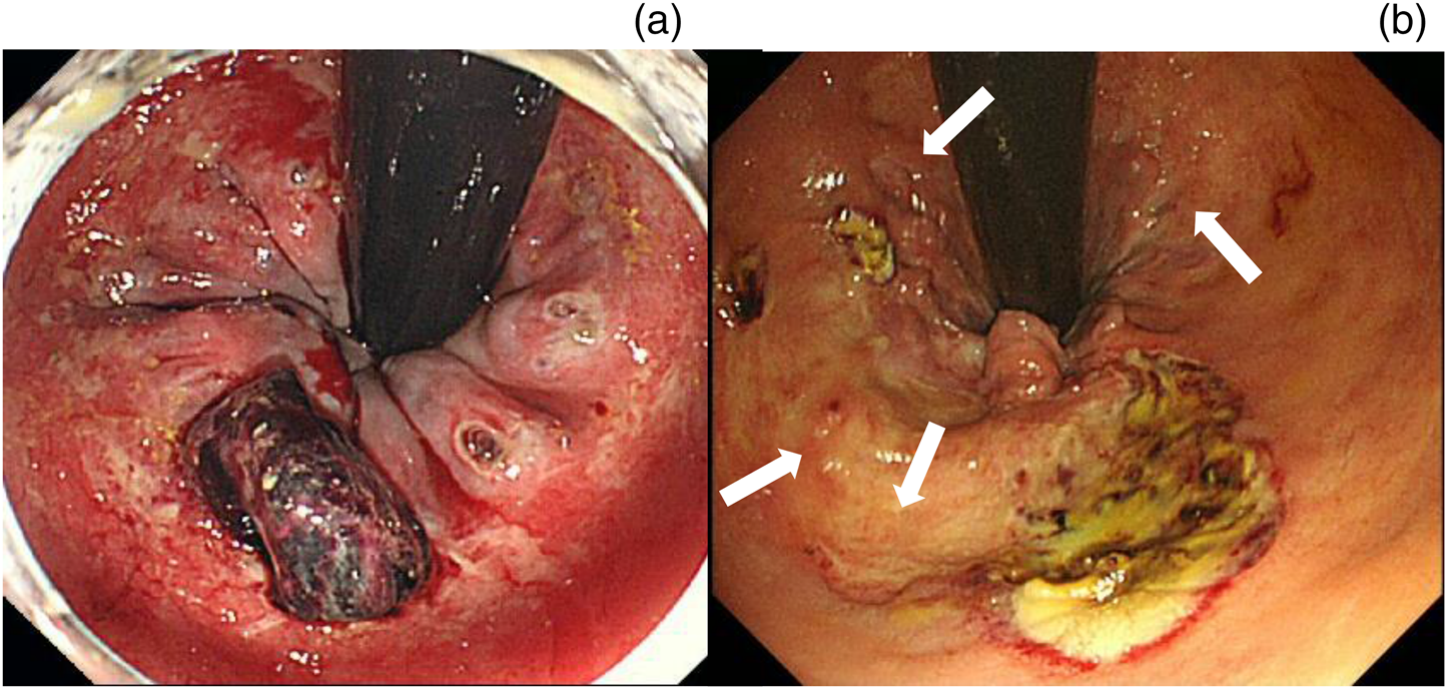
Examples of endoscopic photographs of cases of acute hemorrhagic rectal ulcer syndrome. (a) A case of a Dieulafoy lesion with multiple exposed vessels. (b) A case in which rebleeding occurred 14 times; several ulcer scars (arrows) are visible around the new ulcer

A high rate of rebleeding is critical in managing patients with AHRUS. The risk factors for rebleeding are under investigation, and no consensus has been reached in the literature. In this study, active bleeding during initial endoscopy was a significant risk factor for rebleeding, even when endoscopic hemostasis was successful. It is unclear why active bleeding at the first emergency endoscopy increases the risk of rebleeding. From our observations, plausible explanations could include poor healing of ulcers due to poor general health conditions, a larger diameter of the ruptured blood vessel, and arterial hemorrhage. One crucial observational finding in our study was the presence of bleeding from multiple vessels (Figure 6a) or the occurrence of rebleeding in an area other than the initial bleeding point.

Reportedly, circumferential‐type ulcers are a risk factor for rebleeding,^14^ and it is plausible that bleeding points can frequently develop in a circumferential ulcer more than in a small ulcer. It is challenging to perform pretreatment in bedridden patients with poor general health conditions, especially those in shock. Moreover, it is difficult to establish a complete field of vision while aspirating residual stool and blood clots. Even in such a situation, the entire rectum, or as much as possible, should be observed, considering the possibility of multiple bleeding points. Since there was bleeding from the new ulcers, another measure to prevent rebleeding is fundamentally recovering the patients’ general conditions.

### Difficulties in identifying Dieulafoy lesions

Dieulafoy lesions are reportedly associated with a high rebleeding rate and are characterized by abnormally large, exposed vessels with small, shallow ulcers.^24^ However, because of the ambiguity in defining ulcer size, abnormal vascular lesions that do not form ulcers may be reported as Dieulafoy lesions. Therefore, we herein classified all lesions without ulceration as Dieulafoy lesions as it was difficult to distinguish between Dieulafoy and vascular lesions.

In our study, there was no significant difference in the rebleeding rates between Dieulafoy and floor‐forming lesions. Based on our clinical experience, once a bleeding point can be identified, CLIP is effective for the hemostasis of the exposed bleeding duct in a Dieulafoy lesion as it is surrounded by normal mucosa. However, the problem is the difficulty in identifying Dieulafoy lesions. Thirty‐five patients were observed without hemostasis as the bleeding point could not be identified at the initial endoscopy; 11 of these patients experienced rebleeding. At least four patients had active bleeding at the second or subsequent endoscopy; six of these could not note the bleeding point even at the second endoscopy; hemostatic treatment could not be performed (Table 2). In most of the 11 cases, ulcer lesions were found; however, since it was challenging to identify the bleeding point even after multiple observations, we believe that bleeding may have occurred from an unidentified Dieulafoy lesion. Identifying elusive bleeding points is a severe issue that requires further investigation.

### Endoscopic hemostasis

CLIP hemostasis methods induced significantly less rebleeding. Only the exposed blood vessel is gripped directly to stop the bleeding in CLIP; the destruction of the surrounding tissue is minimal. Contrarily, EC involves coagulation of the mucous membrane around the broken blood vessel, and the destruction of the surrounding tissue in EC is larger than that of CLIP. Therefore, the observed difference in rebleeding rates between the methods is reasonable as EC may have exacerbated the hypoperfusion of the rectal mucosa and delayed wound healing by damaging the tissue. However, since this is a retrospective observational study with a limited number of patients, treatment selection or patient background bias cannot be definitively excluded. The endoscopists’ skills may greatly influence CLIP. At the facility where the current study was conducted, at least two endoscopists oversaw all examinations. Hemostatic procedures were performed under the supervision of a senior physician, thus ensuring a certain level of quality was maintained. The provision of stable clipping techniques may be one factor contributing to this study's low clipping rebleeding rate.

EC requires effective electrosurgical hemostats and an electrosurgical generator with appropriate power and effect settings to utilize the appropriate coagulation mode for the organ and situation. In this study, the settings of the electrosurgical generator were set at the endoscopist's discretion. Furthermore, different modes of coagulation waves and types of hemostatic forceps may slightly reduce tissue damage.

CLIP had no significant difference in the univariate analysis, whereas it had a significant difference in the multivariate analysis; this is due to active bleeding, which showed a significant difference even in the univariate analysis and could be stopped by CLIP. Of the 40 patients with active bleeding at the initial endoscopy, the 21 who did not rebleed received various treatments (CLIP: 13, EC: 2, COM: 5, endoscopic band ligation: 1), with CLIP being the most common. Of the 53 patients with no active bleeding at the initial endoscopy, 10 had hemostasis with clips and no rebleeding (Table 5). In other words, clipping is effective regardless of the presence or absence of active bleeding. Therefore, endoscopists should avoid using EC and stop the bleeding with CLIP in patients with AHRUS.

### Lesson learned case

One of the study participants experienced 14 episodes of rebleeding due to AHRUS (Figure 6b). Repeated hemostasis performed through EC induced scarring of the surrounding mucous membranes, resulting in an intractable ulcer. Thus, CLIP was used in place of EC. The ulcer healed over an extended period as the patient's general condition improved. However, CLIP became difficult after scar tissue had formed, suggesting that the order in which treatment methods are selected is crucial.

### Limitations

This study had some limitations. First, this was a single‐center study with a limited number of patients. Second, the retrospective study design may have caused treatment selection and patient background biases. Third, the endoscopists’ skill and equipment quality may have affected the efficacy of endoscopic hemostasis and the degree of tissue damage. Therefore, additional multicenter prospective studies with more cases would be desirable.

## CONCLUSION

In bedridden older individuals with poor general health, multiple combinations of arteriosclerosis‐related factors and protracted wound healing factors can induce AHRUS. We strongly recommend performing hemostasis via CLIP on suspected bleeding points, including active bleeding sites, in AHRUS.

## CONFLICT OF INTEREST

None.

## Data Availability

The data supporting this study's findings are available on request from the corresponding author, Natsumi Uehara. However, the data are not publicly available as they could compromise the privacy of research participants.
